# Effect of an orthodontic resin modified with silver-nanoparticles on enamel color change 

**DOI:** 10.4317/jced.59224

**Published:** 2022-03-01

**Authors:** Marco Sánchez-Tito, Lidia-Yileng Tay

**Affiliations:** 1 Facultad de Estomatología, Universidad Peruana Cayetano Heredia, Lima, Peru; 2 Facultad de Ciencias de la Salud, Universidad Privada de Tacna, Tacna, Peru

## Abstract

**Background:**

To evaluate the effect of an orthodontic resin modified with various concentrations of silver-nanoparticles (AgNPs) on enamel color.

**Material and Methods:**

Twenty lower premolars were collected and divided into four groups (n = 5) according to the concentration of AgNPs (0.05%, 0.1%, 0.5%, and 1% wt/wt). Additionally, a group bonded with a conventional resin was used as control (n=5). Previous to the bracket bonding, enamel color was measuring with a spectrophotometer. Specimens were stored for 6 months in deionized water. Brackets were debonded and color changes of enamel were calculated with the CIEDE 2000 formula (∆E00). One-way ANOVA and Tukey test were used to assess the color change (α = .05).

**Results:**

The control group showed the lowest ∆E00 values, and no significant difference was observed when compared to the group with 0.05% of AgNPs (*P* > .05). The highest color change was observed in the group with 1% of AgNPs (∆E00 = 8.04±1.48), however no significant difference was observed between groups with 0.1% and 0.5% AgNPs.

**Conclusions:**

The incorporation of AgNPs into orthodontic resin result in tooth color alteration. Lower concentrations of AgNPs should be considered to minimize negative changes in enamel color.

** Key words:**White spot lesions, antibacterial, orthodontics, adhesive, Silver-nanoparticles.

## Introduction

White spot lesions (WSLs) are one of the adverse effects of orthodontic treatment, and can affect up to 50% of these patients (1,2). These lesions are the result of a process of enamel demineralization produced by the accumulation of biofilm in the regions close to the brackets (3). Clinical situations where poor oral hygiene is associated with a higher consumption of fermenTable carbohydrates will allow an increase in the colonization of acidogenic bacteria, which will lead to a reduction in the pH of the oral environment and promote the development of WSLs (4).

To prevent the occurrence of WSLs, various approaches have been proposed, but these strategies are highly dependent on the patient adherence to treatment (5). Other strategies have sought to incorporate antibacterial agents to orthodontic resins that would inhibit bacterial growth, avoiding the issues related to patient adherence (6,7).

A wide variety of antibacterial agents have been incorporated into the resins (8-13). *In vitro* studies have shown that the incorporation of these agents does not seem to affect the physical and mechanical properties of orthodontic resins (8,9,11), although there are still some controversies regarding this issue (12).

The antimicrobial properties of nanoparticles are widely recognized (10-13). In general, their properties are related to the release of ions, the reduced size of the particles, and the increase of the surface contact area with the bacterial membrane, affecting its function and promoting cell death (14,15).

In previous studies, AgNPs have been incorporated in different concentrations in orthodontic resins and an important antibacterial effect on microorganisms associated with the formation of WSLs has been demonstrated (10,11,13).

Products containing silver ions are effective for caries prevention (16,17); however, when the released free silver ions (Ag+) are reduced, they aggregate, precipitate and penetrate the enamel surface up to 25 µm resulting in the formation of dark stains (18,19), causing esthetic concerns.

Although orthodontic resins with the addition of AgNPs have shown antibacterial properties, there is no information related to the staining effect of these resins on tooth enamel. Therefore, the present investigation aims to evaluate the effect on enamel color change of an orthodontic resin modified with various concentrations of silver-nanoparticles. The null hypothesis was that the incorporation of different concentrations of silver-nanoparticles in an orthodontic resin does not affect enamel color.

## Material and Methods

-Ethical consideration and sample size calculation

This study was approved by the Local Ethical Committee. The calculation of the sample was carried out in the G*power 3.1 software, using the comparison of multiple means of a previous study (20), where the tooth-staining effect of addition of AgNPs in a dental material was evaluated. An effect size of 2.25, an error of 5% and a power of 90% were adopted. The minimum sample size calculation was 4 specimens per group. Finally, in this study it was decided to include twenty-five sound human lower premolars extracted for orthodontic reasons. The teeth were cleaned and stored in distilled water at 4°C for no longer than 90 days. The specimens were randomly assigned to the study groups (n = 5). Orthodontic brackets were bonded using an orthodontic resin modified with 0.05%, 0.1%, 0.5% and 1% of AgNPs, and five additional teeth were bonded with a conventional orthodontic resin (control group).

-Experimental orthodontic resin preparation

A light-cured orthodontic resin (Transbond XT; 3M Unitek, Monrovia, CA, USA) was used to incorporate AgNPs (US Research Nanomaterials, Houston, TX, USA, average particle size: 20 nm, purity: 99.99%) at different concentrations (0.05%, 0.1%, 0.5% and 1%). The resin was mixed with the AgNPs using a plastic spatula and a glass slab for 2 min in a dark room (13). To obtain the different concentrations, 3 g of the resin was mixed with the corresponding quantity of AgNPs (wt/wt). The resins were stored in sterilized black syringes at room temperature until their use.

-Bracket bonding procedure 

**Figure 1:**
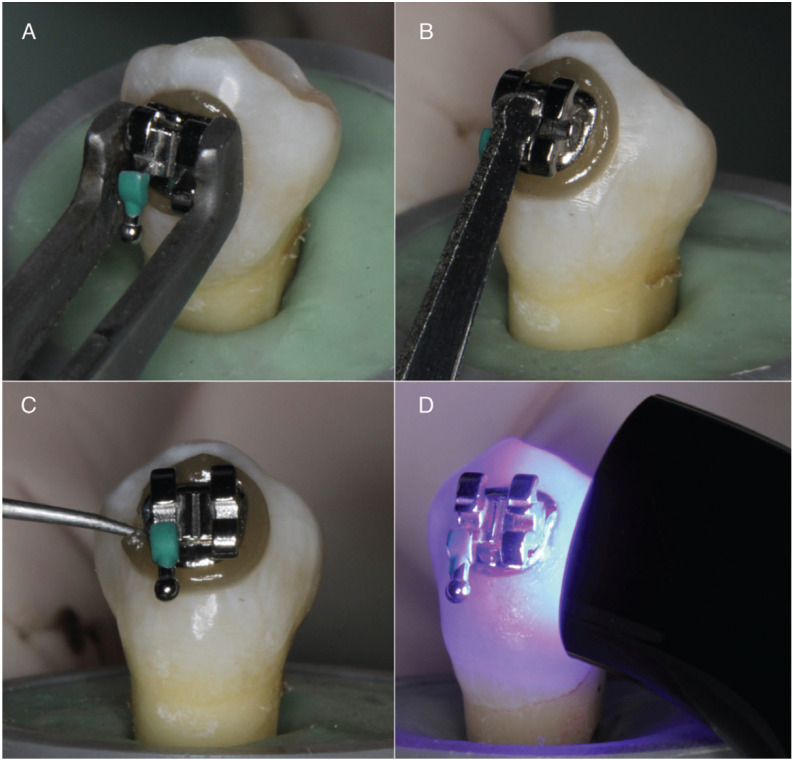
Detail of the bracket bonding process with the modified resin with silver-nanoparticles. (A) Bracket positioning. (B) Constant force to standardize the resin thickness. (C) Removal of excess resin. (D) Light-curing por 40 seconds.

All the teeth were polished with pumice for 30 seconds and washed with deionized water. Brackets were bonded at the center of the clinical crown (9). 37% phosphoric acid (Unitek Etching Gel, 3M, Monrovia, CA, USA) was applied for 30 seconds, washed with deionized water for 1 min and air-dried (8,9). The primer (Transbond XT Primer; 3M Unitek, Monrovia, CA, USA) was applied with a brush and then light-cured for 20 seconds (Elipar DeepCure-L unit; 3M Unitek, Monrovia, CA, USA) at 450 nm wavelength, and 1470 mw/cm2. Orthodontic brackets (Gemini, 3M Unitek, Monrovia, CA, USA) were bonded with the experimental resins. The resin was applied to the bracket base and it was placed on the tooth. To standardize the resin thickness, a constant force of 250 gf was applied for 20 seconds with a dynamometer gauge (12). Excess resin was removed and the specimens were light-cured for 20 seconds from mesial and distal side of the bracket (8) (Fig. 1). All specimens were stored at 37°C (8) for six months in individual test tubes containing 10 mL of deionized water that was renewed every week.

-Bracket debonding

After storage time, the brackets were carefully removed with an orthodontic plier (Ormco, Glendora, CA, USA). Resin remnant from enamel surface was removed with a scalpel blade. To ensure a resin-free surface, an eighteen-multiblade zirconia orthodontic bur (Dental Morelli, Sorocaba, SP, Brazil) was used for 60 seconds with moderate pressure using a low speed handpiece under copious water cooling.

-Color measuring

The teeth were kept in wet environment before initial color measurements, to avoid color alteration. The color measuring was performed using a digital spectrophotometer (EasyShade V, Vita Zahnfabrik, Bad Säckingen, Germany) previous to the bracket bonding procedure (T1) and after removal of the brackets (T2). Three measurements were recorded in each time and mean values were obtained for each specimen. To ensure the reproducibility of the measurements and the correct positioning of the spectrophotometer tip, a silicone matrix was used (Zetaplus, Zhermack SpA, Italy). Previous to the color measurement, the spectrophotometer was calibrated according to the manufacturer’s instructions.

Data were recorded following the CIE system: L*, a*, and b*. The color change from T1 and T2 was calculated using the CIEDE2000 formula (∆E00), which uses h (hue) and C (Chroma) values (21). In this study the KL, KC, and KH parametric values were set to 1. The color change was considered clinically unacceptable when the values were higher than 3.7 units, according to previous studies (22,23).

-Statistical testing

Statistical analysis was performed using SPSS 22.0 Statistics software (SPSS, Chicago, IL, USA). Data distribution was previously checked for normality with the Shapiro-wilk test. One-way analysis of variance (ANOVA) and Tukey post-hoc multiple comparison test were used to assess the color change values of enamel. All statistical tests were set at a significant level of α = 0.05.

## Results

Color changes of enamel between the groups, according the concentration of AgNPs contained in the orthodontic resin after six months are shown in Table 1. One-way ANOVA test showed significant difference among the color changes in all groups (*P* < .05). Comparing the control group (2.00 ± 0.51) and the group with 0.05% AgNPs (3.31 ± 0.70), the ∆E00 values showed no significant difference, and these values were lower than those of the groups containing 0.1%, 0.5%, and 1% of AgNPs.

**Table 1:**
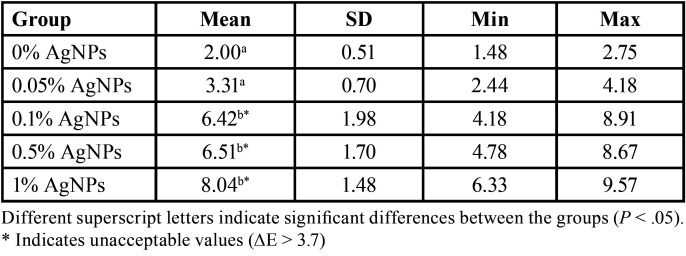
Examination of color change (∆E00) of enamel after debonding of orthodontic brackets.

The highest color change was observed in the group with 1% of AgNPs (8.04 ± 1.48); however, there was no significant difference when multiple comparison was made with groups at 0.5% and 0.1% of AgNPs (*P* > .05).

**Figure 2:**
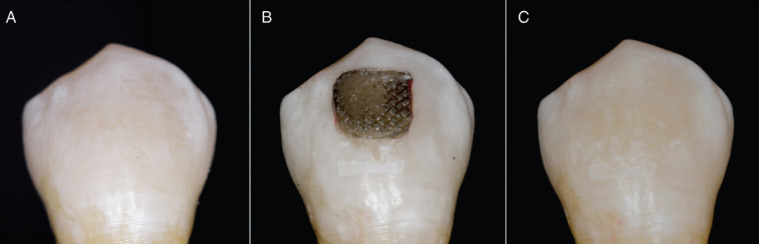
Detail of a specimen. (A) Initial appearance of enamel. (B) Resin after the bracket debonding after six months of storage. (C) Final appearance of enamel after resin removal.

The use of the experimental resin affected the values of L*, a*, and b* coordinates (Table 2). All samples showed negative values in lightness (∆L*); negative values indicate that the samples became darker. No significant differences were observed between the control group and the group with 0.05% AgNPs (*P* > .05), while in the groups with a higher concentration of AgNPs, a greater decrease in lightness was observed, with the greater change in ∆L* observed in the group 1% AgNPs (-10.86 ± 2.65). Furthermore, all samples showed negative values of ∆a*, which means a change towards green color, and there was no significant differences between the groups. When ∆b* was assessed, all samples showed a shift towards blue color, with the greater change seen in the group with 1% AgNPs (-7.50 ± 1.21) (*P* < .05). Figure 2 shows the color change observed in a specimen after resin removal.

**Table 2:**
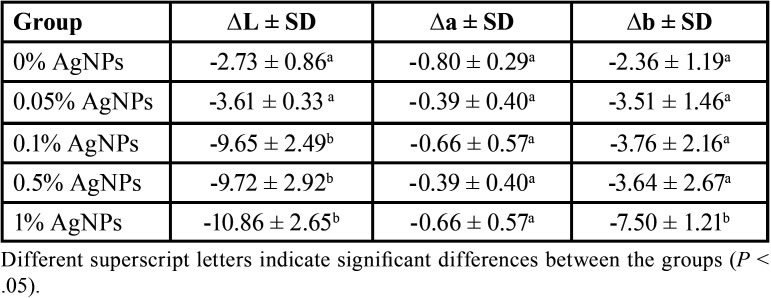
Changes of L*, a*, and b* in the groups with different AgNPs concentrations.

## Discussion

In this study, AgNPs were added to an orthodontic resin at different concentrations and the effect on enamel color change was assessed. The results showed that the use of the experimental resin AgNPs for bracket bonding, affected the color of enamel after six months of storage, and the null hypothesis was rejected.

The use of AgNPs have been extensive employed in the dental field (24,25). Concentrations close to 1% show important antibacterial effects without affecting the mechanical properties of dental materials (13,25). The incorporation of higher concentrations of AgNPs produces color change in the material, causing it to acquire a dark grey color, affecting the esthetic characteristics of the material. Concentrations greater than 1% can increase the esthetic problems (26). In this study, all the concentrations of AgNPs affected the color of the orthodontic resin, producing a grey color of different shades, depending on the increase in the concentration.

In this study, the CIEDE2000 (∆E00) formula was chosen because it is more sensitive and a better indicator to assess acceptability in color changes (27). Color change of enamel was observed in all groups after storage for six months in deionized water, even for the control group. According to a previous study, teeth immersed in distilled water for 24 hours showed a color change due to the sorption of water in the enamel (28), and this could explain why the samples of the control group showed a decrease in the ∆E00 values.

In this study, the acceptable threshold level of ∆E was set at 3.7 units (22,23). Accordingly, color changes below 3.7 were considered acceptable. The results showed that the enamel of the groups with a concentration greater than 0.1% of AgNPs showed ΔE values greater than 3.7 units; therefore, these color changes were considered clinically unacceptable. Nevertheless, there was no significant differences between groups with 0.1%, 0.5% and 1% of AgNPs (*p* > .05). Only the lowest concentration (0.05%) can be considered clinically imperceptible to the human eye, and comparable to the color changes produced in the control group. In a previous study, the use of a concentration of 0.05% of AgNPs in an orthodontic resin has been effective to inhibit the growth of *S. mutans* and *L. acidophilus* (25). Thus, this concentration would be effective to improve the antibacterial properties of orthodontic resins without affecting the color of the enamel.

The incorporation of AgNPs into the orthodontic resin affected the measurement of L*, a*, and b* coordinates. It is known that the values of ΔL* are more significant since they are more easily perceived by the human eye than the Δa* and Δb* values (22). There were no significant differences in ΔL* values in the groups with concentrations greater than 0.1% (*p* > .05). The higher change in ΔL* was observed in the group with 1% of AgNPs (-10.86 ± 2.65), which clinically affected the color of the teeth, making them look darker. It is important to note that this color change may be due to the aggregation and precipitation of silver ions on enamel surface (18,26). Furthermore, the AgNPs used in this study had an average size of 20 nm, which could be related to the observed effect. Horst *et al*., described that the free silver ions can penetrate up to 25 µm on the enamel surface causing its pigmentation (19). Additionally, there was a small change in Δa* in all the groups, and these changes indicates a mild shift towards green color, and could be explained by the storage in the deionized water, which has been shown to affect the color of the teeth (29). Meanwhile the values seen in Δb* express a shift towards blue color. In this regard, it was observed that the higher the percentage of AgNPs used, the greater the change in terms of ∆E00, ∆L*, ∆a* and ∆b*.

Evidence-based research indicates that the enamel after brackets bonding may show color changes, assuming that the compounds of the adhesive systems may produce this effect (30). orekçi *et al*. demonstrated that color alterations can be observed on teeth bonded with different orthodontic resins, and these changes are related to the increase in L* and a* values, while the b* values decreased (31). Zaher *et al*. demonstrated that self-etching primers produce less penetration of resin tags into enamel, resulting in less color change after orthodontic treatment (23). Moreover, in a study by Joo *et al*., it was observed that the use of self-etching primers showed less susceptibility to pigmentation in enamel, after debonding and polishing (32). These findings should be considered when assessing color changes, in addition to the incorporation of AgNPs in the experimental resin since they could affect the observed results.

Hernández-Sierra *et al*. assessed the teeth color change after the use of a combination of a copolymer Gantrez S-97 and AgNPs in the form of toothpaste, and their results showed that the use of this combination did not produce significant changes in enamel color, which could be explained due to the medium size of AgNPs (40 nm to 80 nm) (33). According to these findings, the size of the nanoparticles may be related to their staining potential. This strategy may be applied to characterize orthodontic resins with AgNPs and avoid the negative effect on enamel color; further studies are needed in order to verify their possible use.

As part of the limitations of the study, we can point out that the storage time of the teeth was six months based on a previous study (31). However, this is less than the average time of duration of a conventional orthodontic treatment, which could have an impact on the recorded ∆E values. Consequently, future studies are necessary to evaluate the enamel color change after an evaluation time similar to the duration of orthodontic treatments. On the other hand, *in vitro* studies cannot include other variables associated to the color change of resins and enamel, such as the absorption of staining substances from the diet, since it is know that enamel color can be affected by the instability of the resins to these variables (34).

## Conclusions

The incorporation of AgNPs to an orthodontic resin at concentrations greater than 0.1% affected the color of the enamel after six months of storage. Additionally, the incorporation of AgNPs mainly affected the values of L* coordinate, making the teeth look darker.
